# Supplementary material for: Determinants of Health-Related Quality of Life After Transarterial Chemoembolization in Hepatocellular Carcinoma Patients: A Systematic Review
Source: J Clin Med. 2025 Jun 3;14(11):3941. doi: 10.3390/jcm14113941 (PMC12155868; doi:10.3390/jcm14113941)
Supplement: Supplementary file 1 [file jcm-14-03941-s001.zip › Supplementary Materials S2-Search strategy.pdf]

Seach Strategy

P (population): HCC patients who underwent TACE (HCC patients post-TACE)

I (Intervention/Exposure)：Potential influencing factors on HRQoL (eg, pain, liver function, psychological distress, nutrition, complications)

C(Comparison): Different levels or presence/absence of influencing factors (e.g., high vs. low anxiety, with vs. without pain)

O（Outcome）: HRQoL post-TACE, measured by validated scales (e.g., SF-36, EORTC QLQ-C30, FACT-Hep)

Pubmed: 71 title and abstract

Search: (((((((factor*[Title/Abstract]) OR (influenc*[Title/Abstract])) OR (determinant*[Title/Abstract])) OR (predictor*[Title/Abstract])) OR (correlate*[Title/Abstract])) OR (contributor*[Title/Abstract])) AND (((qol[Title/Abstract]) OR (HRQoL[Title/Abstract])) OR (quality of life[Title/Abstract]))) AND (((TACE[Title/Abstract]) OR (transarterial chemoembolization[Title/Abstract])) OR (chemoembolization[Title/Abstract]))

Scopus: 67 title and abstract

TITLE-ABS ("factor*" OR "determinant*" OR "predictor*" OR "correlate*" OR "contributor*") AND TITLE-ABS ("qol" OR "HRQoL" OR "quality of life") AND TITLE-ABS ("TACE" OR "transarterial chemoembolization" OR "chemoembolization")

Web of Science: 144 title and abstract

(factor* OR determinant* OR predictor* OR influenc* OR correlate* OR contributor*) AND (qol OR "quality of life" OR HRQoL) AND (TACE OR "transarterial chemoembolization" OR chemoembolization)

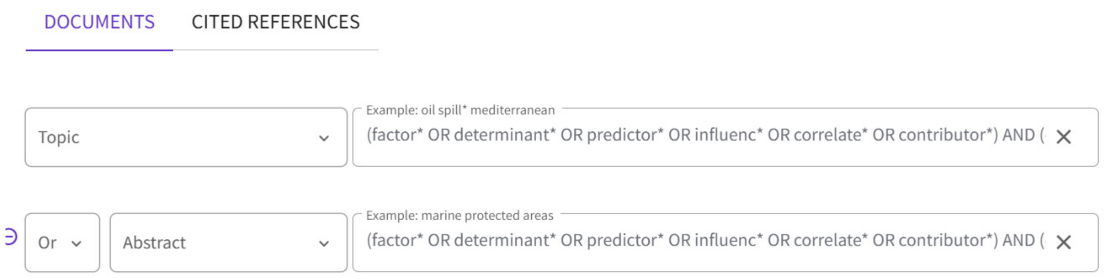

CNKI：　110 title and abstract

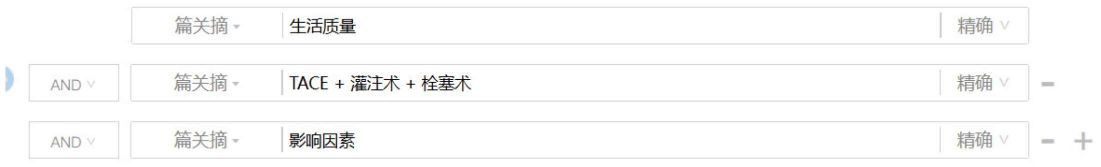

Definition：(factor* OR determinant* OR predictor* OR influenc* OR correlate* OR contributor*) AND (qol OR "quality of life" OR HRQoL) AND (TACE OR "transarterial chemoembolization" OR chemoembolization)

万方：　250 title and abstract

((主题=生活质量) AND 主题=(TACE OR 栓塞术 OR 灌注术)) AND 主题=(影响因素)

Definition: (factor* OR determinant* OR predictor* OR influenc* OR correlate* OR contributor*) AND (qol OR "quality of life" OR HRQoL) AND (TACE OR "transarterial chemoembolization" OR chemoembolization)
